# Photoreduction and validation of haem–ligand intermediate states in protein crystals by *in situ* single-crystal spectroscopy and diffraction

**DOI:** 10.1107/S2052252517002159

**Published:** 2017-04-10

**Authors:** Demet Kekilli, Tadeo Moreno-Chicano, Amanda K. Chaplin, Sam Horrell, Florian S. N. Dworkowski, Jonathan A. R. Worrall, Richard W. Strange, Michael A. Hough

**Affiliations:** a School of Biological Sciences, University of Essex, Wivenhoe Park, Colchester CO4 3SQ, England; b Swiss Light Source, Paul Scherrer Institut, 5232 Villigen-PSI, Switzerland

**Keywords:** X-ray photoreduction, laser photoreduction of haem, haem, *in crystallo* optical spectroscopy

## Abstract

Powerful synergies are available from the combination of multiple methods to study proteins in the crystalline form. Spectroscopies which probe the same region of the crystal from which X-ray crystal structures are determined can give insights into redox, ligand and spin states to complement the information gained from the electron-density maps. The correct assignment of crystal structures to the correct protein redox and ligand states is essential to avoid the misinterpretation of structural data. This is a particular concern for haem proteins, which can occupy a wide range of redox states and are exquisitely sensitive to becoming reduced by solvated electrons generated from interactions of X-rays with water molecules in the crystal. Here, single-crystal spectroscopic fingerprinting has been applied to investigate the laser photoreduction of ferric haem in cytochrome *c*′. Furthermore, *in situ* X-ray-driven generation of haem intermediates in crystals of the dye-decolourizing-type peroxidase A (DtpA) from *Streptomyces lividans* is described.

## Abstract 2

Integrated structural biology can yield powerful synergies and maximize the biological information gained. Two examples are described of combining X-ray crystallography with single-crystal resonance Raman and UV–visible spectroscopies to study the functions of haem proteins.

## 1. Introduction   

High-resolution crystal structures determined using synchrotron radiation are central to our understanding of haem-protein function. It is therefore essential for correct biological interpretation that these structures accurately represent the relevant redox/ligand states of the haem Fe. Metal sites such as haem moieties are exquisitely sensitive to redox (and ligand) state changes caused by X-ray-generated solvated photoelectrons even at cryotemperatures (Beitlich *et al.*, 2007 ▸; Kekilli *et al.*, 2014 ▸; Dworkowski *et al.*, 2015 ▸; Hersleth & Andersson, 2011 ▸) or, in the case of single-crystal Raman spectroscopy, to photoreduction by the excitation laser itself (Kekilli *et al.*, 2014 ▸). In solution resonance Raman (RR) spectroscopy, samples are often translated during data collection to minimize the photoreduction of the susceptible redox centres. An increasing number of synchrotron beamlines now have the capability to measure spectroscopic data *in situ* and online from the crystals used for X-ray crystallographic data collection, providing the capability for effective validation and the unambiguous assignment of individual structures to particular mechanistic steps. Previous studies have exploited X-rays to generate haem-protein species within crystals for structural characterization. In particular, several key intermediates from the catalytic reaction of P450cam were generated by X-ray exposure (*via* redox processes following the X-ray generation of solvated photoelectrons) and structurally characterized (Schlichting *et al.*, 2000 ▸), while similar studies have produced the structures of intermediates in the peroxidase cycle of myoglobin (Hersleth *et al.*, 2007 ▸, 2008*a* ▸,*b* ▸; Hersleth & Andersson, 2011 ▸). In this study, we present the use of on-axis and online single-crystal spectroscopies at beamlines BM30 at the European Synchrotron Radiation Facility (ESRF) and X10SA at the Swiss Light Source (SLS) to fingerprint the oxidation and ligand states of two different haem proteins and to confirm the presence and identity of functionally relevant intermediates. These proteins have previously been studied by solution spectroscopies and the protein crystals diffracted to high resolution, making them ideal systems to couple solution and structural data and to identify and assign the observed oxidation/ligand and intermediate states.

We describe the use of online UV–visible spectroscopy to monitor the X-ray-generated formation of an oxyferrous complex of the dye-decolorizing-type peroxidase A (DtpA) from *Streptomyces lividans* (Petrus *et al.*, 2016 ▸), allowing its structural characterization. Crucially, both the electron-density maps and spectroscopic data were required to identify the intermediates that were present, and differences owing to temperature or sample state were apparent, demonstrating the synergy between the two methods.

The five-coordinate haem protein cytochrome *c*′ from *Alcaligenes xylosoxidans* (AXCP) is able to discriminate between the gaseous ligands NO and CO by binding them on different faces of the haem (distal *versus* proximal) while excluding the binding of O_2_ (Hough & Andrew, 2015 ▸). We have previously shown that high-quality single-crystal resonance Raman (SCRR) data can be measured from AXCP crystals (Kekilli *et al.*, 2014 ▸) together with atomic resolution crystallographic data using the MS3 on-axis single-crystal microspectrophotometer at the SLS. We have also previously described the *SLS-APE* *in situ in crystallo* optical spectroscopy data-analysis toolbox and how to characterize and treat sample-dependent artefacts in a reproducible manner (Dworkowski *et al.*, 2015 ▸). We have also characterized the power-density dependence of laser photoreduction of haem using the common excitation wavelength of 405 nm, which lies within the Soret absorption band. A threshold effect is seen for laser photoreduction, such that at low power densities (1.6–4.0 mW mm^−2^) no photoreduction is observed, while above the threshold (>4.0 mW mm^−2^) photoreduction is time dependent. This work demonstrates the importance of the combination of crystal spectroscopies and macromolecular crystallography to obtain maximum functional information on haem-protein crystals in that important catalytic intermediates with similar electron densities can be differentiated by additional spectroscopic measurements.

## 2. Methods   

Cytochrome *c*′ from *A. xylosoxidans* (AXCP) was expressed, purified and crystallized as described previously (Kekilli *et al.*, 2014 ▸). Crystals of dimensions ∼1.0 × 0.3 × 0.3 mm were transferred into a cryoprotectant solution consisting of 40% sucrose, 2.4 *M* ammonium sulfate, 0.1 *M* HEPES buffer pH 7.5 for 10 s before flash-cooling in liquid nitrogen. SCRR data were measured using the on-axis MS3 instrument at beamline X10SA, Swiss Light Source (Pompidor *et al.*, 2013 ▸). The excitation wavelength was 405.4 nm and the focused laser spot size at the crystal was ∼25 µm. The spectrometer entrance slit width was 100 µm and a 2400 lines mm^−1^ grating blazed at 300 nm was used for dispersion. Raman shifts were calibrated against cyclohexane or 4-acetamidophenol standard samples. Laser powers at the sample position were monitored using a Thorlabs PMD100D hand-held power meter. The sample laser power showed a linear dependence upon the input laser power in the range 50–400 mW (Supplementary Fig. S1). A kinetic series of 20 × 40 s exposures were measured at each sample input power at 100 K with the X-ray shutter closed. SCRR data were measured in the wavenumber range 575–2000 cm^−1^ with particular interest in the porphyrin redox-state and core-size marker regions (1300–1700 cm^−1^). All spectra were processed using the *SLS-APE* analysis package (Dworkowski *et al.*, 2015 ▸). The proportions of ferric and ferrous protein populations in each spectrum were calculated by fitting the spectral range 1340–1380 cm^−1^ to one or two Gaussian peaks (corresponding to each redox state) using the multi-peak analyser tool in *OriginPro* 8 (OriginLab Corporation). The time-dependence of the spectra of the high-density powers (*i.e.* 5.9 and 9.8 mW mm^−2^) were fitted to a single exponential decay function and the lower density powers (*i.e.* 1.6 and 4.0 mW mm^−2^), where no reduction was observed, were fitted to a linear function.

DtpA was overexpressed and purified as previously reported (Petrus *et al.*, 2016 ▸). Crystals were grown at 18°C by the hanging-drop vapour-diffusion method using a ferric protein solution at 13 mg ml^−1^ equilibrated against a reservoir consisting of 17–24% PEG 3000, 50–100 m*M* sodium citrate pH 5.5. Crystals grew within 1–2 d to dimensions of 100 × 50 × 50 µm and were transferred into a cryoprotectant containing the reservoir solution supplemented with 30% sucrose before flash-cooling in liquid nitrogen. Hydrogen peroxide (H_2_O_2_) soaks were prepared by transferring ferric DtpA crystals into a crystallization solution supplemented with 0.5%(*v*/*v*) H_2_O_2_. Single-crystal UV–visible absorption spectroscopy of DtpA crystals was carried out at 100 K using the online microspectrophotometer (von Stetten *et al.*, 2015 ▸) at beamline BM30A (FIP) at the ESRF. The microspectro­photometer was set up in a plane perpendicular to the X-ray beam (McGeehan *et al.*, 2009 ▸). The objectives were positioned at 45° with respect to the φ axis to allow access for the cryostream and for sample changing. A combined deuterium–halogen lamp (DH2000, Ocean Optics) was used as the light source. The microspectrophotometer was focused into a 25 µm spot and it was aligned to probe the same region as the X-ray beam. Spectroscopic data over the absorbance range 200–1000 nm were measured using the *SpeCuBE* software (von Stetten *et al.*, 2015 ▸) and were initially processed using the *SpectraSuite* software (Ocean Optics). Each optical absorption spectrum was the result of ten accumulations of 200 ms exposures.

X-ray crystallographic data for DtpA were measured on BM30A at a wavelength of 0.98 Å using an ADSC Q315r CCD detector. Crystallographic data were indexed, integrated and merged using *XDS* (Kabsch, 2010 ▸) and *AIMLESS* (Evans & Murshudov, 2013 ▸) in the *CCP*4 suite with the *CCP*4*i*2 interface. The structure was initially solved by molecular replacement using *MOLREP* (Vagin & Teplyakov, 2010 ▸) with a DyP structure from *S. coelicolor* as the search model with a sequence identity of 95% (PDB entry 4grc; T. Lukk, A. M. A. Hetta, A. Jones, J. Solbiati, S. Majumdar, J. E. Cronan, J. A. Gerlt & S. K. Nair, unpublished work). The structures were refined in *REFMAC*5 (Murshudov *et al.*, 2011 ▸) and rebuilt between refinement cycles using *Coot* (Emsley *et al.*, 2010 ▸). Structures were validated using *MolProbity* (Chen *et al.*, 2010 ▸), the *JCSG Quality Control Check* server and the PDB validation server. The coordinates and structure factors were deposited in the Protein Data Bank with accession codes 5map and 5mjh. X-ray absorbed doses were calculated using *RADDOSE*-3*D* (Zeldin *et al.*, 2013 ▸).

## 3. Results   

### 3.1. Laser-induced photoreduction of AXCP crystals   

**Figure 1:**
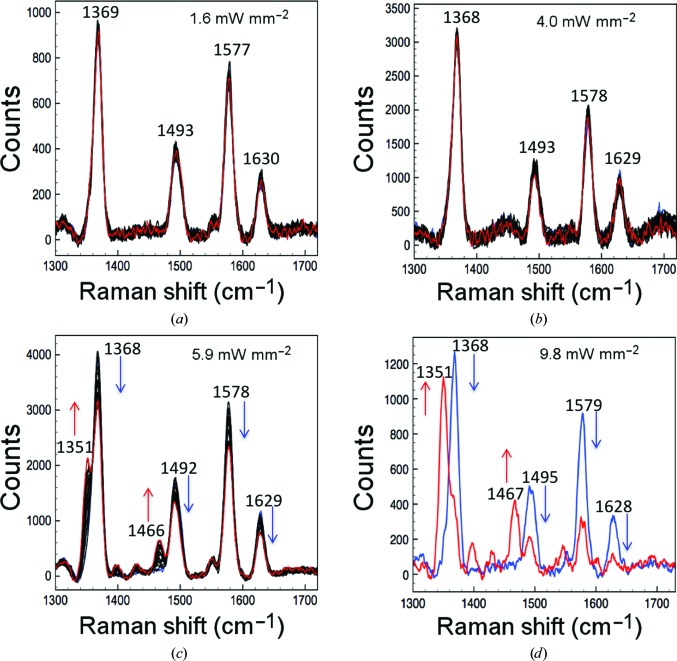
Photoreduction of the ferric haem of AXCP crystals by excitation with a 405 nm laser. (*a*, *b*) No photoreduction is observed at low laser power densities from 1.6 to 4.0 mW mm^−2^ at the start of (blue spectra) and after (red spectra) the kinetic series. (*c*) Higher laser density powers (>4.0 mW mm^−2^) result in clear photoreduction of the Fe, as shown by the split of the ν_4_ marker at 1368 cm^−1^ and interconversion into the 1351 cm^−1^ peak, which is evidence for the ferrous state of the haem Fe. (*d*) This interconversion and the presence of only the 1351 cm^−1^ band are strong evidence of complete reduction and occur more rapidly at higher power densities such as 9.8 mW mm^−2^. The blue spectrum represents the first spectrum of the first kinetic series and the red spectrum represents the last spectrum of the last kinetic series.

We characterized the photoreduction of AXCP crystals from the ferric to ferrous oxidation state by laser irradiation at different laser power densities. SCRR data were measured from ferric AXCP crystals using 405.4 nm laser excitation and laser power densities between 1.6 and 9.8 mW mm^−2^. Initially, 20 sequential RR spectra each of 40 s laser exposure at a laser power density of 1.60 mW mm^−2^ were measured over the wavenumber range 575–2000 cm^−1^. The first spectrum exhibits porphyrin marker bands characteristic of ferric AXCP (Fig. 1 ▸; Supplementary Table S1), consistent with our previous SCRR data (Kekilli *et al.*, 2014 ▸). The final spectrum in the series, with a total laser exposure of 800 s, was essentially identical to the first spectrum, indicating that no laser-induced photoreduction from the ferric to the ferrous state had occurred (Fig. 1 ▸ *a*). A comparison with available solution data further confirmed the ferric redox state of the crystals (Kekilli *et al.*, 2014 ▸), with small shifts of 1 cm^−1^ for the redox-state marker and 4 cm^−1^ for some of the core-size markers. The small differences are within the error of the instrument of approximately ±3 cm^−1^.

**Figure 2:**
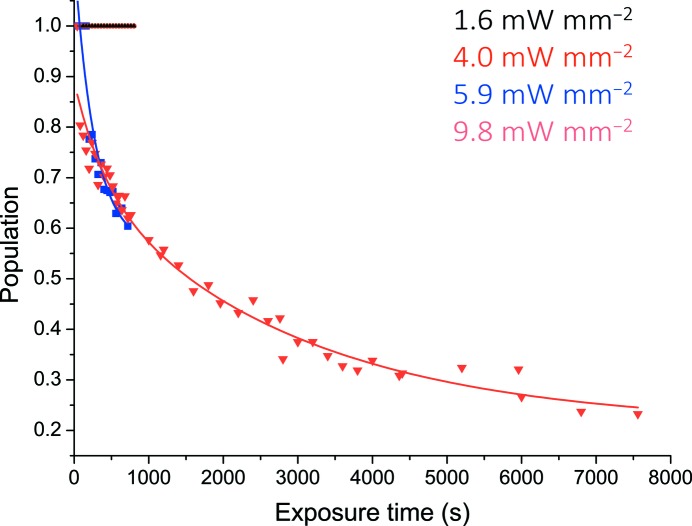
Time-dependent photoreduction of AXCP crystals. Photoreduction was not observed for laser power densities of 1.6 and 4.0 mW mm^−2^. Laser power densities of >4.0 mW mm^−2^ result in reduction of the haem. Data points for power densities where photoreduction was observed have been fitted to a single exponential decay function. Data for an extended exposure time are included for a power density of 9.8 mW mm^−2^ to demonstrate almost complete interconversion to the ferrous form.

The experiment was repeated following a translation of the crystal by 120 µm to bring a nonlaser-exposed region into the beam. A power density of 4.0 mW mm^−2^ again caused no significant spectral changes over 20 exposures and the SCRR spectrum remained characteristic of the ferric state over a total laser exposure of 800 s (Fig. 1 ▸ *b*). At higher laser powers significant changes to the spectrum were observed with increasing laser exposure, consistent with photoreduction to the ferrous state in a time-dependent manner. A small but significant level of photoreduction was observed following the measurement of 20 spectra at 5.9 mW mm^−2^, with a total laser exposure of 800 s (20 × 40 s; Fig. 1 ▸ *c*). The gradual interconversion of the redox-state marker ν_4_ to 1351 cm^−1^ and the disappearance and decrease of other high-intensity bands, for example, the disappearance of the 1492 cm^−1^ band and the appearance of the 1466 cm^−1^ band, is indicative of laser-induced reduction of the haem Fe. A further increase of the laser power density to 9.80 mW mm^−2^ again using a fresh region of the crystal resulted in significantly faster reduction with clear interconversion from the ferric to the ferrous state after only a few spectral measurements, with the majority of the crystal in the reduced state at the end of the kinetic series, after a total laser exposure of 2000 s (Supplementary Fig. S2). At this power density, significant sample reduction within the measurement time of the first spectrum with equal proportions of the ferric and ferrous state occurred at 800 s and rapid interconversion to an almost fully ferrous state occurred after ∼1000 s. The rates of reduction at each laser power density are shown in Fig. 2 ▸, based on the interchange between the measured Raman intensities at ∼1370 cm^−1^ (corresponding to ferric protein) and ∼1351 cm^−1^ (corresponding to ferrous protein). In all cases, the spectra indicate a clear conversion from the ferric to the ferrous state without the formation of intermediate species (isosbestic point at 1359 cm^−1^) and in agreement with solution RR data for the redox-state and core-size markers of the ferrous protein (Supplementary Table S1). The population of ferric and ferrous haem Fe was fitted using linear or exponential decay functions.

### 3.2. Generation and structure determination of validated haem–ligand intermediates in DtpA   

**Figure 3:**
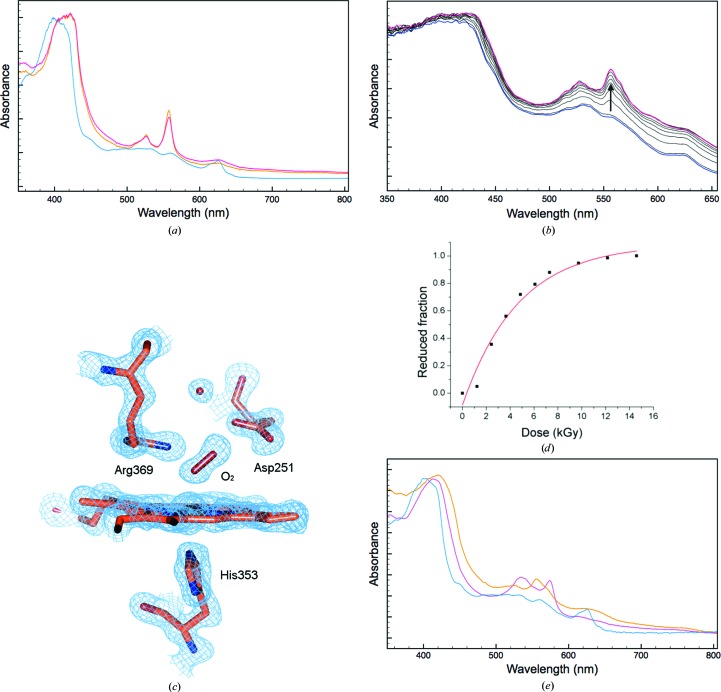
Single-crystal UV–Vis spectroscopic data and X-ray structure of DtpA. (*a*) UV–visible spectra of a DtpA crystal at 100 K. The initial spectrum (blue) is most consistent with a ferric haem state (Table 1 ▸), which is reduced following measurement of the first (orange) and second (red) crystallographic data sets. (*b*) Reduction of the haem followed in small dose (kGy) increments. The starting ferric spectrum in shown in blue, with progressive reduction after 1.2, 2.4, 3.7, 4.9, 6.1, 7.3, 9.7 and 12.2 kGy dose exposure shown in black and the last spectrum after 14.6 kGy shown in magenta. (*c*) Spectroscopically validated crystal structure of the oxyferrous form of DtpA (the haem site of monomer *A*; 2*F*_o_ − *F*_c_ electron density contoured at 1σ). Online UV–Vis spectra were measured before and after collection of the data set, shown in orange and red, respectively, in (*a*). (*d*) Proportion of protein in the ferrous state as a function of accumulated dose. Data points were fitted to an exponential function. (*e*) UV–visible spectrum of a peroxide-treated DtpA crystal (magenta) compared with a ferric crystal spectrum (blue). The spectrum of the peroxide-treated crystal following X-­ray exposure is shown in orange.

Online spectroscopic data collection from a DtpA crystal prior to X-ray exposure gave the blue spectrum reported in Fig. 3 ▸(*a*). The λ_max_ of the Soret band is in good agreement with the ambient solution-state ferric spectrum of DtpA (Table 1 ▸; Petrus *et al.*, 2016 ▸). However, at lower wavelengths the spectrum of the crystal is not as well resolved as in the solution state, where a broad absorbance band with a λ_max_ of 502 nm and a shoulder at 540 nm has been reported (Table 1 ▸; Petrus *et al.*, 2016 ▸). Consistent with the solution spectrum is a band at 624 nm (635 nm in solution) that is indicative of a high-spin ferric haem. Therefore, the observation of this band together with the λ_max_ of the Soret band are strong support for the crystal being in the ferric haem oxidation state. Exposure to X-rays resulted, as expected, in rapid spectral changes that can be associated with reduction of the ferric haem (Figs. 3 ▸ *a* and 3 ▸ *b*). The dose-dependence of this reduction was determined using a 0.3 × 0.3 mm top-hat X-ray beam, resulting in a half-dose for reduction of only 3 kGy (Figs. 3*a* ▸ and 3*d* ▸). The spectrum of the X-ray-reduced crystal using a total dose of 0.99 MGy gave a λ_max_ at the positions reported in Table 1 ▸ (Fig. 3 ▸ *a*, orange). At first glance the α and β bands appear to be identical to those reported in solution for compound II (a ferryl haem species; Fe^IV^=O; Table 1 ▸). However, such a species in solution can only be generated by an initial two-electron oxidation of the haem followed by a one-electron reduction to give the Fe^IV^=O species. Thus, such a species is unlikely to be generated in the X-ray beam starting with a ferric crystal (Fig. 3 ▸ *a*). It is more likely that the spectrum is that of a ferrous form of the protein with the α and β bands consistent with a six-coordinate form. The overall tertiary structure of DtpA will be described in detail elsewhere (Chaplin *et al.*, unpublished work). For the present crystal the 0.99 MGy initial structure has two molecules in the crystallo­graphic asymmetric unit, denoted here as *A* and *B*, and has been determined to 1.45 Å resolution. See Table 2 ▸ for crystallographic data-collection statistics and refinement details and Table 3 ▸ for geometrical values for the haem site of the oxyferrous DtpA structure. An electron-density feature at the distal haem face already suggests the formation of a six-coordinate species (Supplementary Fig. S3), with some extra electron density assigned to a partially populated H_2_O molecule. A further high-resolution crystal structure was determined using a second dose of 0.99 MGy (an accumulated dose of 1.98 MGy) to a resolution of 1.49 Å. This revealed electron density at the distal haem face that now clearly supported the presence of a diatomic molecule, to which a dioxygen molecule was modelled (Fig. 3 ▸ *c*). One O atom is ligated to the haem Fe at adistances of 2.3 and 2.1 Å in monomers *A* and *B*, respectively, as well as making a polar interaction with the distal pocket residue Arg369. The second O atom interacts with the carboxylate side chain of Asp251 (Fig. 3 ▸ *c*) and the Fe—O—O bond angle is 145°. The spectrum of the crystal measured following completion of this data set showed no further changes (Fig. 3 ▸ *a*, red). Therefore, we conclude from the structure and the spectroscopic data that the haem is in a six-coordinate ferrous state with an O atom bound (oxy­ferrous). This interpretation highlights a discrepancy between the ambient solution-state absorbance spectrum of the oxyferrous DtpA and that of the same form in a crystal at 100 K (Table 1 ▸). Such spectral differences could arise from temperature, state (solution/crystal) or a mixture of the two. Examination of the electron-density maps and structures for the two successive DtpA structures (with accumulated doses of 0.99 and 1.98 MGy, respectively) revealed no substantial differences in coordinates or *B* factors and no appreciable signs of specific radiation damage beyond the haem pocket (Supplementary Fig. S4).

**Figure 4:**
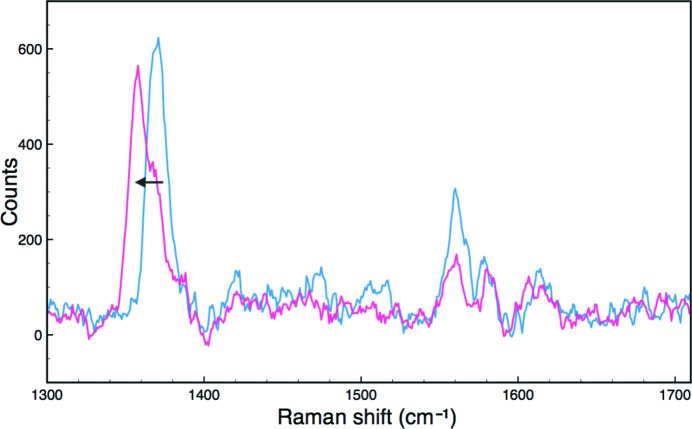
High-frequency single-crystal resonance Raman spectra of DtpA at 100 K. Blue, ferric crystal spectrum before exposure to the X-ray beam; magenta, spectrum of the same crystal after collection of a diffraction data set. A shift in the ν_4_ redox-state marker band (arrow) is consistent with reduction to the ferrous form during data collection.

For comparison of the spectral properties in different mechanistic states, a second crystal of ferric DtpA was soaked in a solution containing 0.5%(*v*/*v*) hydrogen peroxide. The UV–Vis absorption spectrum of the crystal is shown in magenta in Fig. 3 ▸(*e*). This spectrum is similar to the solution spectrum of compound III (an Fe^III^-O_2_^−^ species; Supplementary Fig. S5) and is likely to have been generated from the large excess of H_2_O_2_ added to the crystal. On exposure to X-rays, the spectrum again changes (Fig. 3 ▸ *e*, orange) to one consistent with a ferrous form of the protein that may be a mixture of oxyferrous and deoxyferrous forms. Such behaviour has been observed previously for HRP (Wilmot *et al.*, 2002 ▸), where the reduction of a compound III crystal resulted in a deoxyferrous form. To demonstrate the feasibility of measuring multiple spectroscopic data types from the same batch of crystals, a crystal of DtpA was characterized by SCRR. The spectrum initially showed peaks corresponding to the ferric haem state (Fig. 4 ▸, Table 4 ▸). On X-ray irradiation (total dose 0.15 MGy), the spectrum displayed a clear shift of the haem marker band ν_4_ to 1358 cm^−1^, characteristic of ferrous haem. These frequencies are comparable to those measured from a different DyP in solution and in the crystalline state (Table 4 ▸).

## 4. Discussion   

High-quality online (and on-axis) UV–visible and resonance Raman spectra with good signal to noise were measured from haem protein crystals. The continuous application of low laser powers to AXCP crystals resulted in no significant laser-induced photoreduction, demonstrating that it is feasible both to measure high-quality Raman spectra and to accumulate multiple spectra to improve the signal to noise by extensive averaging. Photoreduction appears to occur only above a certain laser threshold rather than as a result of a direct laser power dose-dependence. This observation differs markedly from the process of X-ray-induced photoreduction of iron(III) to iron(II), which is dose-dependent at any dose rate. While laser photoreduction of ferric haem and porphyrin is a well known phenomenon in solution resonance Raman spectroscopy, the rationale for the observed threshold effect in crystals is not yet clear. The likely mechanism of photoreduction involves ligand–metal charge transfer from the axial Fe ligand. It has been suggested from studies of porphyrins that light photoreduction is often masked by rapid haem reoxidation by O_2_ in aerobic solution samples (Sato *et al.*, 1992 ▸). Photoreduction is more evident in frozen solutions than in liquid solutions, which is presumed to be the result of photoreduced molecules freely diffusing out of the irradiated volume in the latter case (Loehr & Sanders-Loehr, 1993 ▸). In crystals at 100 K, oxygen and other potential oxidizing molecules are immobilized, and only solvated electrons are typically mobile (O’Neill *et al.*, 2002 ▸). Furthermore, such an effect would be expected to only protect against photoreduction until the concentration of O_2_ in the crystal was depleted while, in our data, no photoreduction was observed at lower laser powers upon prolonged exposure. An additional possibility, which has yet to be tested, is that the rate of heat loss from the crystal is relevant, with this being insufficient to prevent photoreduction at higher power densities.

Laser-induced reduction of redox-protein crystals during resonance Raman measurements is a significant problem. Laser reduction in SCRR experiments is particularly insidious as the Raman laser probe has only a limited penetration depth into the crystal surface (∼20 µm; Kekilli *et al.*, 2014 ▸). Laser photoreduction effects may result in a spectral signature for this region which is not representative of the major part of the crystal exposed to X-rays during crystallographic data collection. In this sense, resonance Raman spectroscopy can be used to ensure that no laser-induced changes are occurring during measurement and to monitor X-ray effects on crystals, but cannot be used to drive the protein crystal to a desired redox or catalytic state for structure determination. Using ‘safe’ laser powers that do not in themselves cause changes to the crystals being probed, resonance Raman spectroscopy provides a powerful tool to monitor X-ray-driven reduction effects. In this case, the Raman probe samples a region of the crystal that has been exposed to X-rays and as such can identify the spectral signature associated with a crystal structure. This approach benefits from the on-axis geometry of the spectrometer, as this ensures good alignment of the laser and the X-ray beam, and the measured data, for both spectroscopy and X-ray collection, are taken from the same portion of the crystal. Future studies will address the temperature-dependence of the apparent laser power density threshold effect on laser-induced photoreduction of crystalline haem proteins and seek to provide a mechanistic explanation for this phenomenon.

For crystals of DtpA, clear differences were apparent in the spectra of different haem states between room-temperature solution and 100 K structures. The structures allowed the assignment of 100 K spectra to either a diatomic (oxygen) or monatomic (compound III) state, while the spectra allowed X-ray and ligand-soaking effects to be precisely monitored. In this case, the two methods were highly synergistic in application. The very short lifetimes of ferric haem species and the peroxide-generated species even at 100 K presents challenges for the successful structure determination of functionally relevant complexes and intermediates. Careful spectroscopic validation of such structures is essential in order to optimize the production and structural characterization of such X-ray-sensitive complexes. Obtaining multiple types of spectroscopic data from crystals of the same protein and morphology can be challenging. Thin, plate-like crystals give the clearest UV–Vis absorption spectra, while high optical density cuboid crystals give the strongest SCRR signal. For the examples presented here, the AXCP crystals were essentially opaque and optical absorption data could not be obtained, while the SCRR data were of high quality. For the smaller and less optically dense DtpA crystals, optical spectroscopy was highly effective, while weak but clearly interpretable SCRR data were obtained. For some applications, the growth of crystals of variable sizes and morphologies under conditions that are as similar as possible may be the optimal strategy.

## Supplementary Material

PDB reference: DtpA, 5map

PDB reference: 5mjh

Supporting Information: Supplementary Figures and Tables.. DOI: 10.1107/S2052252517002159/be5277sup1.pdf

## Figures and Tables

**Table 1:** Peak values from UV–Vis spectra measured for different DtpA species both in solution and in the crystalline form Crystal measurements were collected at 100 K and solution data at 293 K.

	Soret band (nm)	β and α bands (nm)	CT band (nm)	Reference
Ferric (solution)	406	502	635	Petrus *et al.* (2016 ▸)
Ferric (crystal)	404	532, 560	624	This work
Oxyferrous complex (solution)	420	539, 571	635	Petrus *et al.* (2016 ▸)
Oxyferrous complex (crystal)	416	526, 557	627	This work
Compound II (solution)	419	528, 557	621	Petrus *et al.* (2016 ▸)
Compound III (solution)	416	541, 577	—	Petrus *et al.* (2016 ▸)
Putative compound III (H_2_O_2_-treated crystal)	415	535, 574	—	This work
Putative deoxyferrous form (X-ray-irradiated crystal)	420	525, 556	624	This work

**Table 2:** Data-collection and refinement statistics for the oxyferrous DtpA structure obtained on beamline BM30 at the ESRF using an X-ray wavelength of 0.98 Å and an ADSC Q315r CCD detector Data sets 1 and 2 represent successive X-ray structures collected from the same DtpA crystal together with single-crystal UV–Vis spectroscopy. Values in parentheses are for the outer resolution shell.

	Data set 1	Data set 2
Space group	*P*2_1_	*P*2_1_
Unit-cell parameters (Å, °)	*a* = 59.78, *b* = 70.63, *c* = 77.64, β = 93.0	*a* = 59.79, *b* = 70.63, *c* = 77.66, β = 93.0
Resolution (Å)	77.53–1.45 (1.47–1.45)	46.16–1.49 (1.52–1.49)
*R*_merge_	0.107 (0.749)	0.096 (0.776)
Unique reflections	112162 (5645)	103431 (5165)
〈*I*/σ(*I*)〉	8.0 (1.6)	8.0 (1.3)
CC_1/2_	0.994 (0.468)	0.993 (0.509)
Completeness (%)	98.3 (99.6)	98.3 (99.7)
Multiplicity	2.9 (3.0)	2.9 (3.0)
*R* factor (%)	22.05	21.92
*R*_free_ (%)	25.42	24.85
R.m.s.d., bond lengths (Å)	0.014	0.015
R.m.s.d., bond angles (°)	1.68	1.74
Ramachandran favoured (%)	97.0	97.0
Accumulated dose (MGy)	0.99	1.98
PDB code	5mjh	5map

**Table 3:** Geometry values for selected bonds and angles at the haem active site of the oxyferrous DtpA structure

	Monomer *A*	Monomer *B*
Fe—N^∊^ His353 (Å)	2.2	2.1
His353 N^δ^—O^δ2^ Asp412 (Å)	2.6	2.6
Fe—O_1_ (Å)	2.3	2.1
O_1_—O_2_ (Å)	1.3	1.3
O_1_—NH_1_ Arg369 (Å)	2.8	3.0
O_2_—O^δ1^ Asp251 (Å)	2.9	3.2
Fe—O_1_—O_2_ (°)	146	144

**Table 4:** Single-crystal high-frequency resonance Raman (SCRR) data for ferric and ferrous DtpA Frequencies for PpDyP and BsDyP are from RR spectra from single crystals at pH 7.3 and 6.5, respectively. Frequencies for DtpA are from single crystals at pH 5.5. *T*, temperature.

Protein	State	*T* (K)	Excitation * λ* (nm)	ν_4_ (cm^−1^)	ν_3_ (cm^−1^)	ν_2_ (cm^−1^)	ν_10_ (cm^−1^)	Reference
SlDtpA	Ferric	100	405.4	1371	1475	1560	1614	This work
SlDtpA	Ferrous	100	405.4	1358	—	1561	1607	This work
PpDyP	Ferric	293	413	1376	1502	1572	1636	Sezer *et al.* (2013 ▸)
PpDyP	Ferrous	293	413	1358	1480	1563	—	Sezer *et al.* (2013 ▸)
BsDyP	Ferric	293	413	1370	1481	1563	—	Sezer *et al.* (2013 ▸)
BsDyP	Ferrous	293	413	1355	1480	—	—	Sezer *et al.* (2013 ▸)
