# Supplementary material for: Photoreduction and validation of haem–ligand intermediate states in protein crystals by in situ single-crystal spectroscopy and diffraction
Source: IUCrJ. 2017 Apr 10;4(Pt 3):263–70. doi: 10.1107/S2052252517002159 (PMC5414400; doi:10.1107/S2052252517002159)
Supplement: Supplementary file 1 [file m-04-00263-sup1.pdf]

IUCrJ

Volume 4 (2017)

Supporting information for article:

Photoreduction and validation of haem-ligand intermediate states in protein crystals by *in situ* single-crystal spectroscopy and diffraction

Demet Kekilli, Tadeo Moreno-Chicano, Amanda K. Chaplin, Sam Horrell, Florian S. N. Dworkowski, Jonathan A. R. Worrall, Richard W. Strange and Michael A. Hough

Table S1 High-frequency single-crystal resonance Raman laser-induced photoreduction measurements on AXCP crystals at 100K using an excitation wavelength of 405.4 nm.

Laser reduction effects were evident above the laser power density of 4.0 mW mm^{-2}. Total laser reduction of the haem from Fe^{3+} to Fe^{2+} within the duration of the experiment was observed at the laser power density of 9.8 mW mm^{-2}.

Laser power densities (mW mm^{-2})	*Time (s)*	*ν_4 (cm^{-1})*	*ν_3 (cm^{-1})*	*ν_2 (cm^{-1})*	*ν_{10} (cm^{-1})*
	0	1369	1493	1577	1630
1.6	800	1369	1493	1577	1630
	0	1368	1493	1578	1629
4.0	800	1368	1493	1578	1629
	0	1368	1492	1578	1629
5.9	800	1368 (1351sh)	1492 (1466sh)	1578	1629
9.8	0	1368	1495	1579	1628
	2000	1351 (1367sh)	1467 (1495sh)	1579	1628

Abbreviations: sh; shoulder

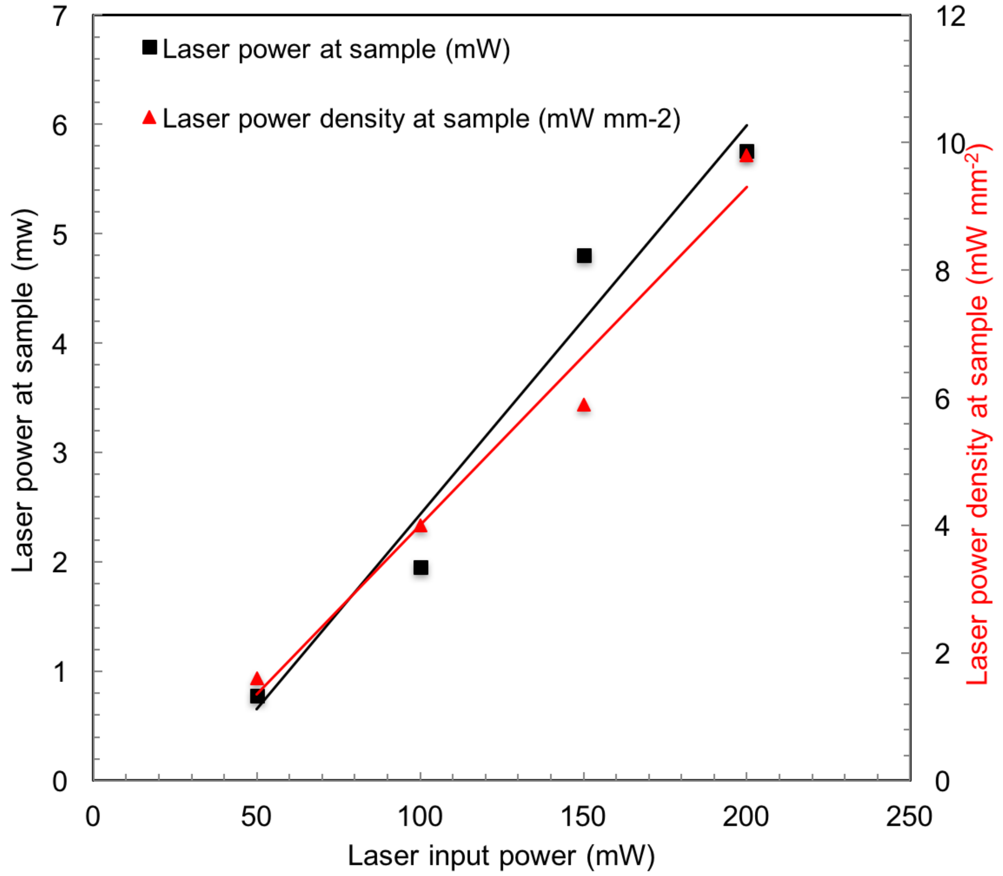

Figure S1 Dependence of measured input laser power and sample laser power at sample position upon the output power of the laser in the optics enclosure. A linear dependence is observed within the measured input laser power from 50 to 200 mW and the laser power densities. (1.6 – 9.8 mW mm^{-2}). The laser power density at each laser power at sample was calculated using the online calculator by OPHIR Photonics. A continuous wave, top-hat laser profile with a diameter of 25 µm was selected.

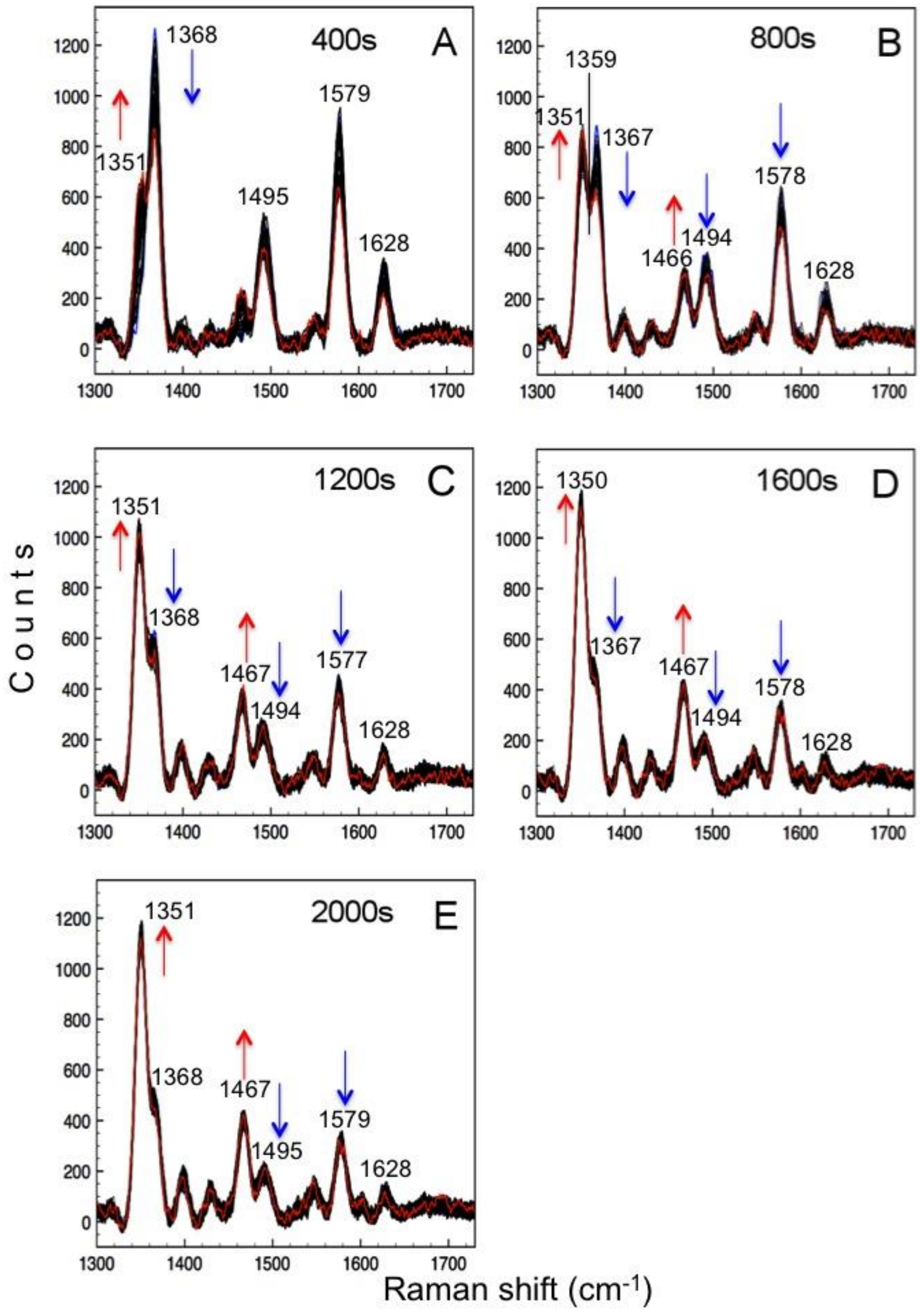

Figure S2 The effects of haem Fe photoreduction on AXCP protein crystals by the excitation laser with a laser power density of 9.8 mW mm^{-2}. (A) Small but significant photoreduction (appearance of the 1351 cm^{-1} shoulder) is evident after the first kinetic series with a total exposure time of 400s; (B) At longer exposures (800s) an equally proportioned ferrous and ferric state is observed with an isosbestic point at 1359 cm^{-1}. (C-E) Clear photoreduction is evident from 1200s and is almost complete by 2000s with the 1351 cm^{-1} peak being the major peak. Red spectra represent the final spectra of each series and blue spectra represent the first spectra of each series with the black spectra being the intermediates.

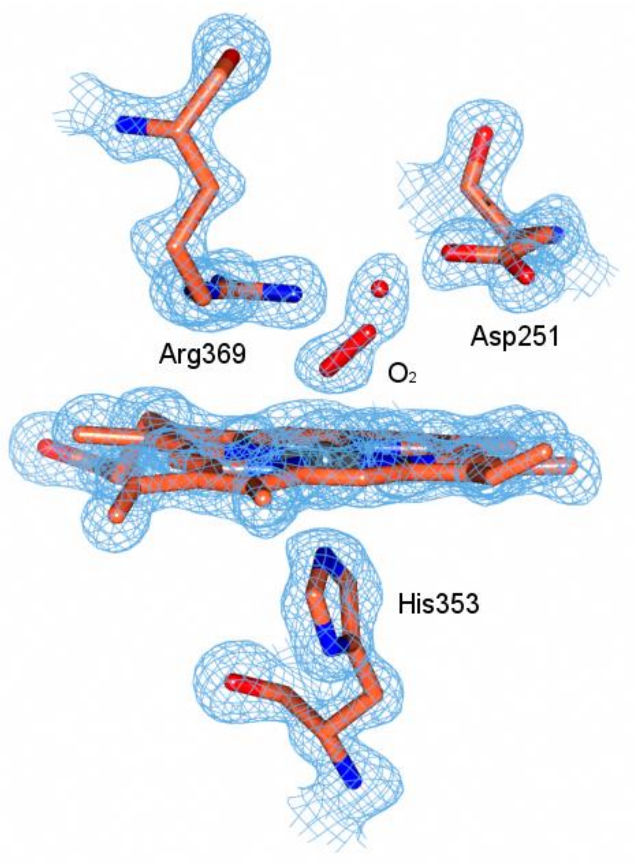

Figure S3 2Fo-Fc map contoured at 1σ of the DtpA haem site of monomer A after collection of the first dataset at BM30 (ESRF), with a dose of 0.99 MGy. Electron density features at the distal site are well fitted with a molecular oxygen molecule. Additional electron density was modelled as a partial occupancy water molecule, stabilized by hydrogen bonding by Asp251.

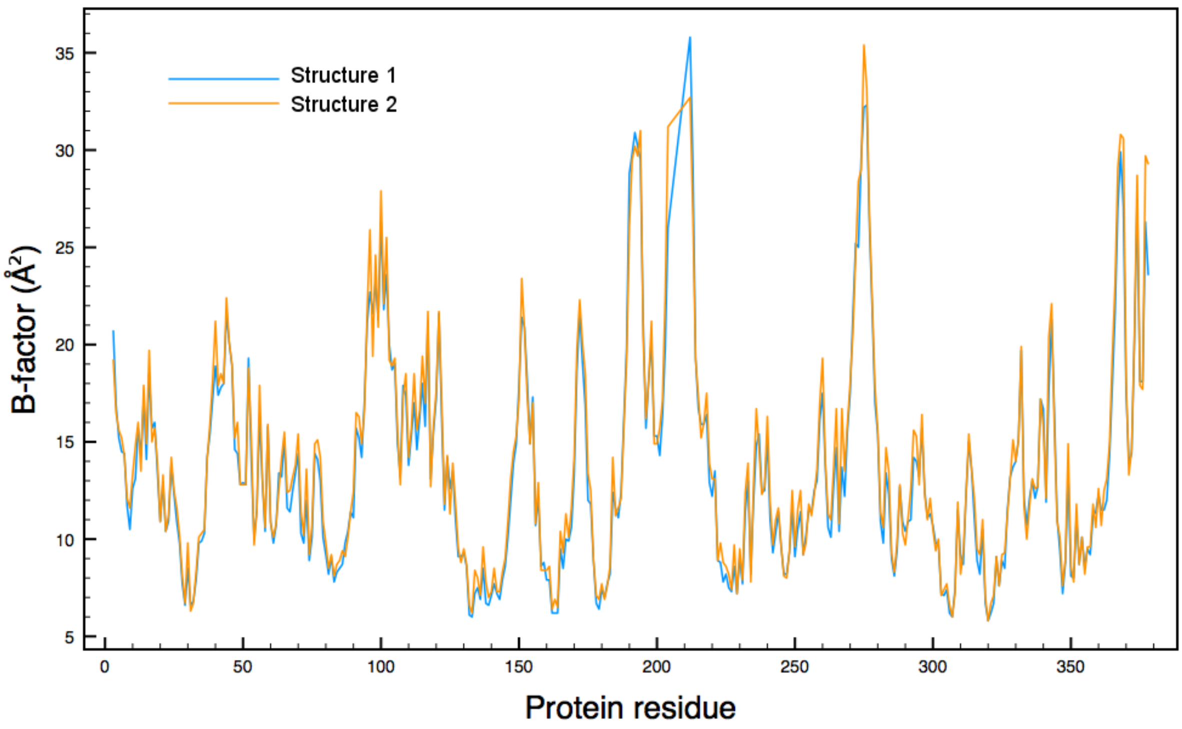

Figure S4 Average all-atom B-factors per residue for the first (0.99 MGy, blue) and second (1.98 MGy, orange) structures of DtpA.

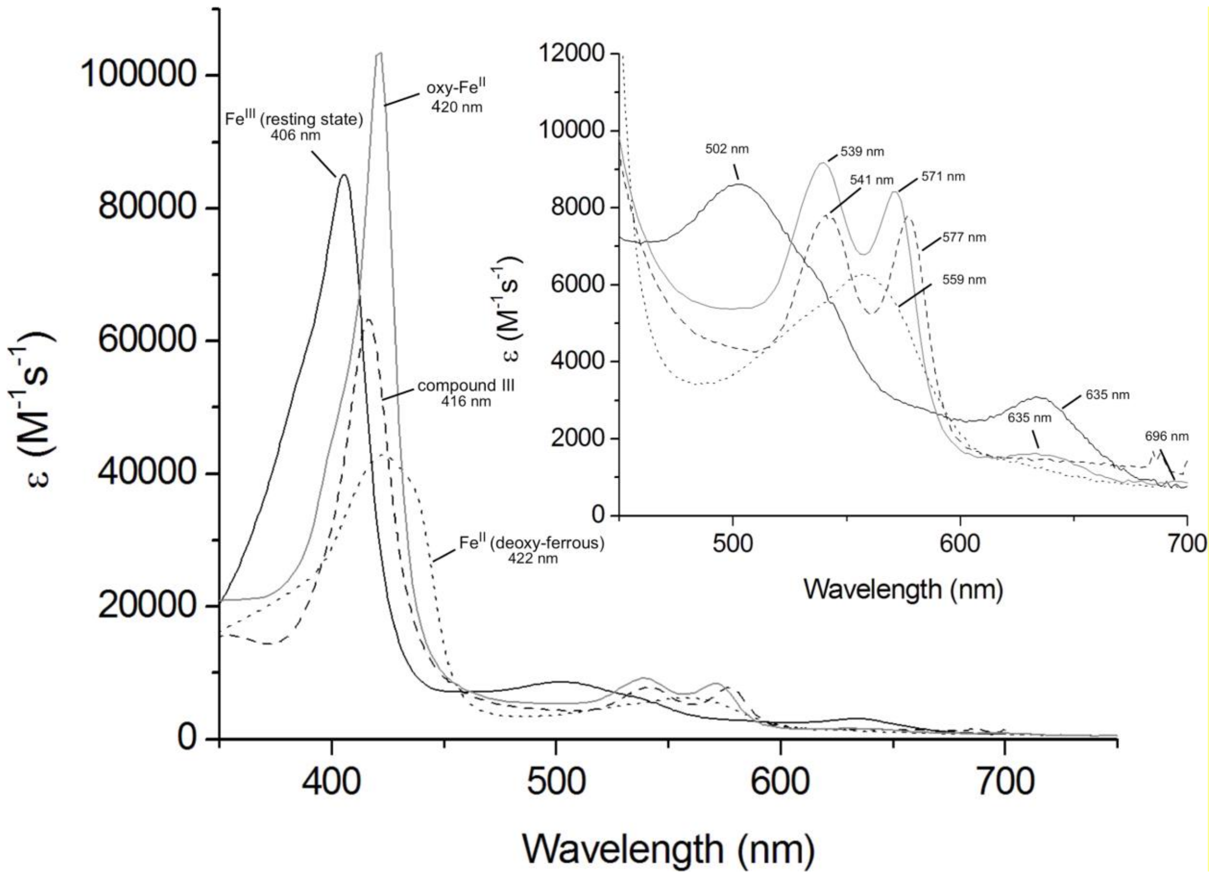

Figure S5 UV-visible absorbance spectra at 20 ºC of various haem oxidation states of DtpA (8 μM in 20 mM NaPi, pH 7, 100 mM NaCl). Resting state ferric (solid black line), compound III (dashed line) generated following addition of 50-fold excess H_2O_2 to the resting state enzyme, deoxy-ferrous (dotted line) following addition of excess $Na_2S_2O_4$ to the resting state enzyme and oxy-ferrous (light grey line) in the presence of glycolaldehyde (a reducing agent). Inset, shows a close up of the Q bands with the relevant wavelengths depicted.
